# *Euterpe oleracea* Mart.-Derived Polyphenols Protect Mice from Diet-Induced Obesity and Fatty Liver by Regulating Hepatic Lipogenesis and Cholesterol Excretion

**DOI:** 10.1371/journal.pone.0143721

**Published:** 2015-12-02

**Authors:** Paola Raquel B. de Oliveira, Cristiane A. da Costa, Graziele F. de Bem, Viviane S. C. Cordeiro, Izabelle B. Santos, Lenize C. R. M. de Carvalho, Ellen Paula S. da Conceição, Patrícia Cristina Lisboa, Dayane T. Ognibene, Pergentino José C. Sousa, Gabriel R. Martins, Antônio Jorge R. da Silva, Roberto S. de Moura, Angela C. Resende

**Affiliations:** 1 Department of Pharmacology, Institute of Biology, State University of Rio de Janeiro, Rio de Janeiro, Brazil; 2 Department of Physiological Sciences, Institute of Biology, State University of Rio de Janeiro, Rio de Janeiro, Brazil; 3 Department of Pharmacy, Federal University of Pará, Belém, Pará, Brazil; 4 IPPN, Federal University of Rio de Janeiro, Rio de Janeiro, Brazil; INRA, FRANCE

## Abstract

The aim of this study was to investigate the effect of a polyphenol-rich Açaí seed extract (ASE, 300 mg/kg-1d-1) on adiposity and hepatic steatosis in mice that were fed a high-fat (HF) diet and its underlying mechanisms based on hepatic lipid metabolism and oxidative stress. Four groups were studied: C57BL/6 mice that were fed with standard diet (10% fat, Control), 10% fat + ASE (ASE), 60% fat (HF), and 60% fat + ASE (HF + ASE) for 12 weeks. We evaluated the food intake, body weight gain, serum glucose and lipid profile, hepatic cholesterol and triacyglycerol (TG), hepatic expression of pAMPK, lipogenic proteins (SREBP-1c, pACC, ACC, HMG-CoA reductase) and cholesterol excretion transporters, ABCG5 and ABCG8. We also evaluated the steatosis in liver sections and oxidative stress. ASE reduced body weight gain, food intake, glucose levels, accumulation of cholesterol and TG in the liver, which was associated with a reduction of hepatic steatosis. The increased expressions of SREBP-1c and HMG-CoA reductase and reduced expressions of pAMPK and pACC/ACC in HF group were antagonized by ASE. The ABCG5 and ABCG8 transporters expressions were increased by the extract. The antioxidant effect of ASE was demonstrated in liver of HF mice by restoration of SOD, CAT and GPx activities and reduction of the increased levels of malondialdehyde and protein carbonylation. In conclusion, ASE substantially reduced the obesity and hepatic steatosis induced by HF diet by reducing lipogenesis, increasing cholesterol excretion and improving oxidative stress in the liver, providing a nutritional resource for prevention of obesity-related adiposity and hepatic steatosis.

## Introduction

Metabolic syndrome (MS) is a disease composed of different risk factors such as obesity, type 2 diabetes, hypertension or dyslipidemia [1, 2]. The prevalence of this syndrome is increasing worldwide in parallel with the rise in obesity. Nonalcoholic fatty liver disease (NAFLD) is now the most frequent chronic liver disease in western countries, affecting more than 30% of the general population. NAFLD encompasses a spectrum of liver manifestations ranging from simple steatosis to steatohepatitis, fibrosis and cirrhosis, which may ultimately progress to hepatocellular carcinoma. There is accumulating evidence supporting an association between NAFLD and MS. Indeed, NAFLD is recognized as the liver manifestation of MS [1]. In particular, abdominal fat accumulation plays an important role in the associated deleterious effects of excess body fat, including dyslipidemia and hepatic steatosis. Although the complex relationship between visceral fat accumulation and hepatic steatosis is not completely understood, dysregulation of lipid metabolism in liver and adipose tissue is associated with adiposity and hepatic steatosis [3].

The liver plays a key role in fatty acid and cholesterol homeostasis because it controls the supply and removal pathways. Adenosine-monophosphate-activated protein kinase (AMPK), a key enzyme of energy metabolism, regulates glucose and lipid uptake, storage and utilization in adipose tissue and liver [4]. AMPK is phosphorylated and then inactivates metabolic enzymes involved in fatty acid (FA) and cholesterol synthesis, such as acetyl-CoA carboxylase (ACC) and 3-hydroxy-3-methylglutaryl CoA reductase (HMG-CoA reductase) [5]. The reduction of pAMPK may play a major role in the pathogenesis of NAFLD [6]. In addition, SREBP-1c (sterol-regulatory-element binding protein-1c) regulates the expression of genes involved in hepatic triacyglycerol (TG) synthesis, and its increased expression [6]. In contrast, the liver eliminates excess of cholesterol from the body either by its conversion into bile acids or after direct secretion into bile by the ATP-biding cassette, subfamily G transporters (ABCG), ABCG5 and ABCG8 [7].

There is increasing evidence supporting an important role to increased oxidative stress in the manifestations associated with obesity [8]. Mitochondrial dysfunction, as a result of uncontrolled oxidative stress, might be a key factor in the pathogenesis of NAFLD with the consequent hepatocellular apoptosis and necrosis [9, 10]. Several studies have shown that treatment with insulin-sensitizing agents [11,12,13], and antioxidants [11, 14] may be useful and might improve the clinical and histological features of nonalcoholic steatohepatitis. However, there is not enough evidence-based support from randomized clinical trials, and the long-term benefit of these medications has not been demonstrated.

Recently, many food components have been studied for their ability to prevent obesity disorders. *Euterpe oleracea Mart*, also known by the popular name of açaí is widely diffused in Amazon region and its fruits are rich in polyphenolic content [15]. We previously reported that açaí seed extract (ASE), rich in catechin and polymeric proanthocyanidins [16] induced endothelium-dependent vasodilatation [17], antihypertensive and antioxidant effects in experimental hypertension [18] and a beneficial effect on metabolic syndrome [19]. However, the mechanism of the antiobesity effect of ASE still remain unclear, and no studies have determined the intrinsic mechanism of ASE on liver lipid metabolism in response to a high fat diet.

In this study, we investigated whether seed extract of açaí, rich in polyphenols could reduce HF-diet-induced obesity and hepatic steatosis in C57BL/6 mice and elucidated its mechanisms.

## Materials and Methods

### Preparation of the acaí seed extract (ASE)

*Euterpe oleracea* Mart. (açaí) fruits were obtained from the Amazon Bay (Belém do Pará, Brazil; excicata number 29052, Museu Goeldi-Belém do Pará). Hydro-alcoholic extracts were obtained from a decoction of the seed. Approximately 200 g of açaí seed were boiled in 400 ml of water for 5 min, grounded for 2 min, and then boiled for an additional 5 min. The decoction was cooled to room temperature, and then extracted by addition of 400 ml of ethanol with shaking for 2 h. The extract was stored in dark bottles at 4°C for 10 days. After this maceration period, the hydro-alcoholic extract of açaí was filtered through Whatman filter paper, and the ethanol was evaporated under low pressure at 55°C. The extract was then lyophilized at -30 to -4°C in a vacuum of 200 mmHg, and then frozen at -20°C until use. Typically, 100 g of seed yielded approximately 5 g of lyophilized extract. The content of polyphenols in ASE, measured by analyzing for total phenol was around 265 mg/g of extract.

### Chemical analysis of ASE

One gram of the lyophilized extract was suspended in 50 mL of distilled water and the resulting solution was extracted with ethyl acetate (3 x 50 mL). After solvent evaporation, the water (95% mass yield) and ethyl acetate (5% mass yield) residues were analyzed by HPLC-DAD on a RP-18 column according to the method developed by Peng et al. (2001) [20]. The ethyl acetate fraction residue was analyzed by negative mode Electrospray Mass Spectrometry after direct infusion on an Ion-Trap Amazon SL spectrometer (Bruker Daltonics).

The mean degree of polymerization (mDP) of the aqueous fraction residue was determined by acid hydrolysis followed by condensation with phloroglucinol, according to the methodology of Kennedy & Jones [21]. The degree of polymerization was confirmed by HPLC-UV on a Diol stationary phase as reported by Kelm et al (2006) [22] and by MALDI-TOF analysis on an Autoflex Speed spectrometer (Bruker Daltonics) according to methodology proposed by Mateos-Martín et al. (2012) [23] with modifications. Briefly, a 1 mg/mL acid solution of the residue of the aqueous fraction was prepared using 0.1% aqueous TFA. Aliquots of the solution were mixed with the matrix (2,5-dihydroxybenzoic acid, 10mg/mL) and the cationic reagent (NaCl, 1mg/mL)to a final mass proportion of 10:1:1 (matrix, sample and cationic reagent, respectively), applied to the target plate and left to dry at room temperature. Mass software was used to analyze the spectral data.

### Animals and Diet

This study was carried out in strict accordance with the recommendations in the conventional guidelines for experimentation with animals (National Institutes of Health Publication No. 85–23 revised, 1996). The protocol was reviewed and approved by the Animal Care and Use Committee of the Institute of Biology of the State University of Rio de Janeiro (protocol n° CEA/025/2010). Male mice of the C57BL/6 strain were obtained from the facilities of the Rio de Janeiro State University at 4 weeks of age. After 1 week of adaptation and at 5 weeks of age, the animals were housed in individual cages in a temperature-controlled room with 12-hour light/dark cycle and randomly allocated into 4 groups. The control group was fed with a standard diet and was allowed access to water (control group: 10% fat; 8.4±0.2 kcal) or ASE (ASE group, 300 mg/kg-1d-1, intragastric gavage). Two other groups were fed a HF diet with access to water (HF group: 60% fat; 12.4±0.3 kcal) or ASE (HF + ASE group: 60% fat; 12.4±0.3 kcal) for 12 weeks (Table 1). The dose of ASE was based on previous studies that showed significant changes in the components of metabolic syndrome induced in HF group [19]. The chronic treatment (12 weeks) with ASE was based on the period of time necessary to induce the metabolic alterations in the C57BL/6 mice fed a HF diet [19]. The diets were elaborated by Rhoster (São Paulo, Brazil) in accordance with the standard recommendations for rodents in the maintenance state of American Institute of Nutrition (AIN-93M) [24].

**Table 1:** Composition and energy content of standard and high-fat (HF) diets.

Nutrient (U/kg diet)	Diets	
	Standard	HF
Casein (g)	140	190
Cornstarch (g)	620.7	250.7
Sucrose (g)	100	100
Soybean oil (g)	40	40
Lard or oil (g)	-	320
Fiber (g)	50	50
Mineral Mix* (g)	35	35
Vitamin Mix * (g)	10	10
L-cystine (g)	1.8	1.8
Choline (g)	2.5	2.5
Antioxidant (g)	0.008	0.008
Energy (kcal)	3800	5400
Percentage as carbohydrate	76	26
Percentage as Protein	14	14
Percentage as Fat	10	60

*Vitamins and minerals mix following the AIN-93M recommendation for rodents

### Food Consumption and Body Weight Measurements

Food consumption of the mice was estimated by subtracting the amount of food left on the grid and amount of spilled food from the initial weight of food supplied. Energy intakes were calculated on the basis of 3.8 kcal/g for the control diet and 5.4 kcal/g for the HF. Body weight was measured weekly and expressed as initial weight (grams) before treatment and week 12 weight (grams) at the end of treatment.

### Serum and liver Assays

After 12 weeks of treatment and at 17 weeks of age, the animals were fasted for 6 hours, and blood samples were then collected by cardiac puncture in anesthetized (thiopental sodium, 70 mg/Kg i.p) animals, after which the animals were euthanized using carbon dioxide. Glycaemia was determined with a glucometer (Accu-Chek Active; Roche, Mannheim, Germany). The contents of serum total cholesterol (TC) and TG were determined by a colorimetric assay (Bioclin, Belo Horizonte, Brazil). Plasma leptin and adiponectin levels were determined by an enzyme-linked immunosorbent assay (ELISA) (Millipore, Missouri, EUA).

The epididymal and retroperitoneal fat mass and liver were removed from animals and weighted. Fragments of the liver of each animal were frozen at– 80°C for further biochemical analysis. Frozen mouse liver (50 mg) was placed in an ultrasonic processor with 1 ml of isopropanol. The homogenate was centrifuged at 2000g and 5 μl of the supernatant was used with a kit for measuring TG and cholesterol in a semi-automatic biochemical analyser (Bioclin, Belo Horizonte, Brazil).

### Western Blot analyses

The total hepatic proteins were extracted in a homogenizing buffer with protease inhibitors. After the liver protein content was detected, the homogenates were centrifuged at 3200 g for 20 min at 4°C, and the supernatants were collected. Equal quantities of total protein were suspended in SDS-containing sample buffer, heated for 5 min at 100°C and separated by SDS-PAGE. After electrophoresis, aliquots (30 μg) of the proteins were electroblotted on to PVDF transfer membranes (Hybond-P; GE Healthcare) and visualized with Ponceau solution staining. The membrane was blocked with phosphate buffer solution plus tween 10 (0.1%) and albumin (5%) and incubated overnight with rabbit polyclonal immunoglobulin G raised against SREBP-1, pACC, ACC, HMG-CoA reductase, pAMPK, ABCG5, ABCG8 and Tubulin antibodies (1:1,000, Santa Cruz Biotechnology, Santa Cruz, CA, USA). β-actin served as a loading control for cytosolic proteins. Following the incubation with the primary antibody, the membranes were washed and incubated with an anti-(rabbit IgG) secondary antibody. Protein expression was detected using an ECL advanced Western blotting detection kit and ECL Hyperfilm (GE Healthcare). The signals were visualized by ChemiDoc Resolutions System and determined by quantitative analysis of digital images of gels using Image-Pro Plus version 7.0 (Media Cybernetics).

### Determination of oxidative damage: carbonyl protein and malondialdehyde assay

Protein carbonylation was determined from the formation of carbonyl group by reaction with 2,4-dinitrophenylhydrazine (2,4-DNPH) according to the method described by Levine et al. (1990) [25].

The lipid membrane damage was determined by formation of products of lipid peroxidation (malondialdehyde-MDA) concentration, using the thiobarbituric acid reactive substances method. Briefly, the samples were mixed with 1 ml of 10% trichloroacetic acid and 1 ml of 0.67% thiobarbituric acid. They were then, heated in a boiling water bath for 30 min. The absorbance of the organic phase containing the pink chromogen was measured spectrophotometrically at 532 nm. MDA equivalents were expressed in nanomole per milligram protein.

### Determination of antioxidant enzyme activity

Superoxide dismutase (SOD), catalase (CAT), and glutathione peroxidase (GPx) activities were assayed in liver homogenates of mice. SOD activity was determined by measuring the inhibition of adrenaline auto-oxidation as absorbance at 480 nm [26]. CAT activity was measured in terms of the rate of decrease in hydrogen peroxide at 240 nm [27]. GPx activity was determined by monitoring the oxidation of NADPH at 340 nm in the presence of hydrogen peroxide [28].

### Histological analysis of the liver

Random fragments of the liver were prepared for light microscopy. Small pieces of liver were embedded in Paraplast plus (Sigma-Aldrich), sectioned at 5 μm and then stained with haematoxylin and eosin. The liver sections were analyzed using light microscopy (Olympus BX 60, Japan) to investigate hepatocyte steatosis and the structure of the hepatic lobules. The images were acquired using Image-Pro Plus version 7.0 (Media Cybernetics) with a sample size of at least 75 fields per group (Olympus DP71, Japan).

### Statistical Analyses

Values are expressed as the mean ± standard error of the mean. Statistical significance was determined by a one-way analysis of variance, with Bonferroni and Tuckey post hoc test. P values less than or equal to 0.05 were accepted as statistically significant.

## Results

### Chemical Composition of ASE

**Fig 1:**
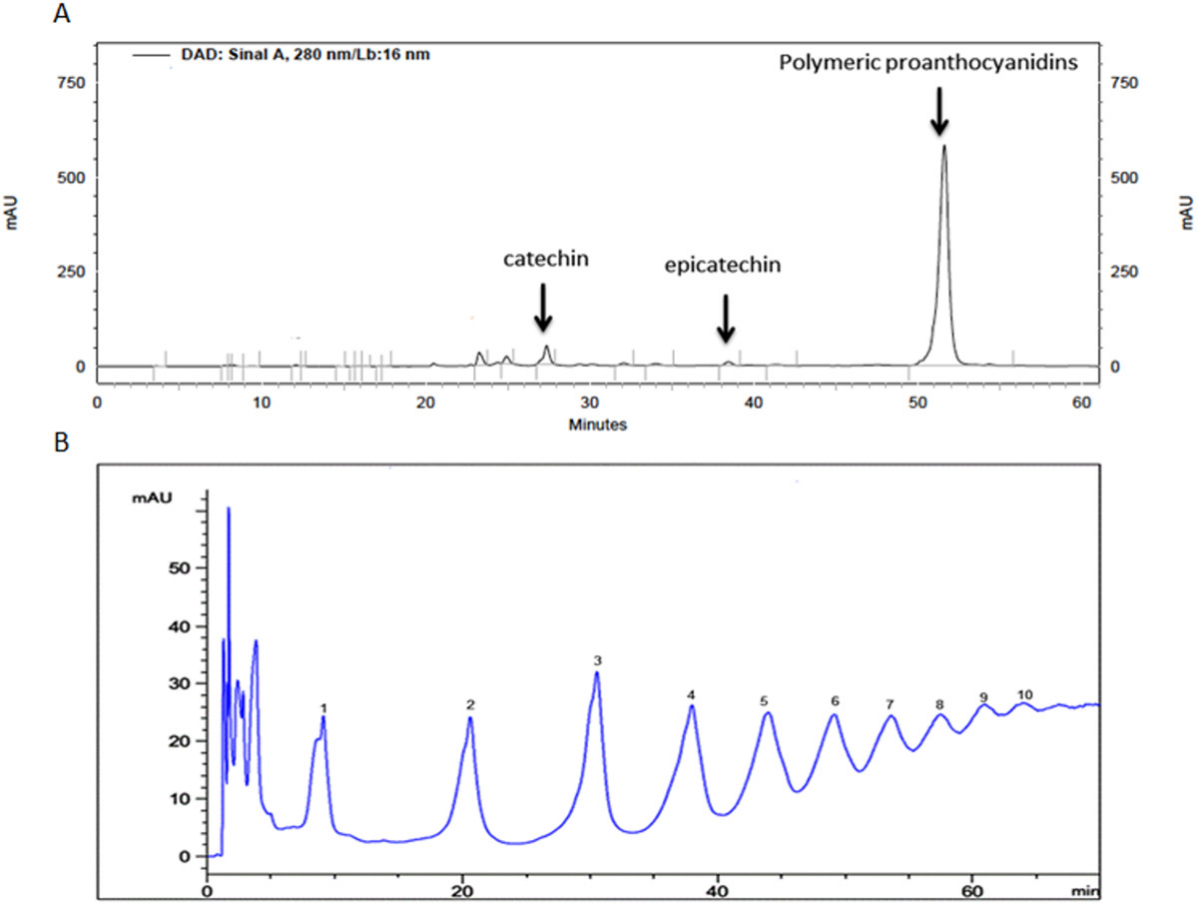
HPLC analysis of ASE. (A) HPLC analysis of the aqueous residue of ASE according to Peng, et al (2001) [21]. The marked peaks of 27, 38 and 51 min correspond to catechin, epicatechin and oligomeric and polymeric condensed tannins. (B) HPLC analysis of the aqueous fraction residue according to Kelm, et al (2006) [23], where the marked peaks (1 to 10) shows the series of condensed tannins present in the sample from monomer to decamer.

Fig 1A shows the chromatogram of the water fraction residue. The big peak eluting last (51 min) displays UV spectral characteristics of condensed tannins and contains predominantly polymeric condensed tannins, proanthocyanidins. The integrated area of this peak is equivalent to 88% of the total area, showing that the extract is composed basically by proanthocyanidins. The peaks at 27 and 38 min are of catechin and epicatechin, respectively, and were identified after co-injection with reference standards. Direct infusion negative ESI MS analysis of the ethyl acetate fraction residue revealed the presence of catechin, epicatechin, a dimer and a trimer at m/z 289, 577 and 865 respectively.

Further experiments were performed to delineate the composition of the main fraction of ASE, the water residue: acid depolymerization in presence of phloroglucinal led to the determination of the mDP as 6.7 and normal phase chromatography was used to assess the extent of polymerization allowing the detection of tannin monomers to decamers (Fig 1B).

**Fig 2:**
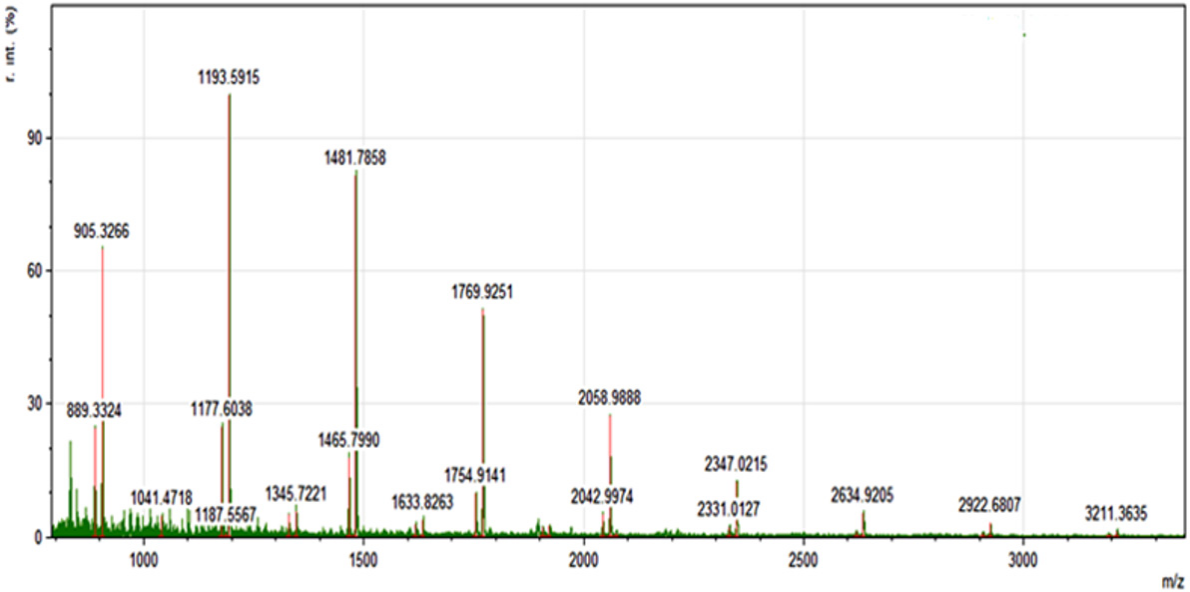
MALDI-TOF mass spectrum of the aqueous fraction residue from ASE. The spectrum was obtained on a Bruker Autoflex Speed; DHB matrix, 1:1:10 (sample: NaCl:matrix). Mass peaks are of sodium adducts of B-type proanthocyanidins: [M + Na] + m/z = 889, 903, 1177, 1193, 1465, 1481, 1753, 1769, 2041, 2058, 2329, 2347, 2617, 2634, 2907, 2922, 3193 and 3211.

MALDI TOF MS has proved to be highly suited to the analysis of the highly complex proanthocyanidin samples [29]. The MALDI TOF MS spectrum of the water residue sample (Fig 2) has shown two major B-type proanthocyanidin peak sequences: the first one of procyanidins (from trimer to undecamer) at m/z (sodium adducts): 889, 1177, 1465, 1753, 2041, 2329, 2617, 2907 and 3193. The second sequence of signals presented peaks separated by 16 mass units from the previous one indicating heteropolymerization with introduction of one unit of (epi) galocatechin from the trimer to undecamer, peaks at m/z (sodium adducts): 905, 1193, 1481, 1769, 2058, 2347, 2634, 2922 and 3211). 3-O-galloylated series were also detected with procyanidin peaks added by multiples of 152 mass units: monogalloylated trimer, mono, di and trigalloylatedtetramer; mono, di and trigalloylatedpentamer all detected in very small amounts. Application of MALDI TOF MS in this field has been critical to unravel the structural complexities of proanthocyanidin samples [30].

Thus, the application of chemical and spectrometric methodology to the analysis of the composition of ASE revealed that it is composed predominantly by polymeric procyanidins, heteropolymers with one gallocatechin unit and, in minor extent, of galloylatedprocyanidins.

### Effect of ASE on Food Intake and Body Weight gain

**Fig 3:**
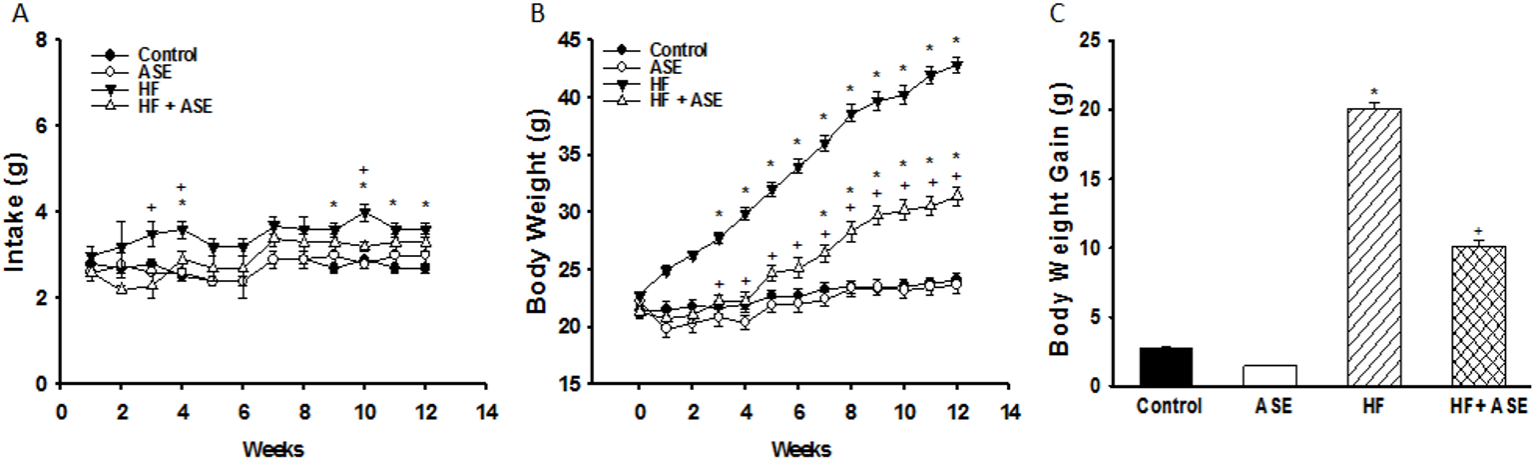
Food intake and body weight. Effects of ASE on food intake (A) and body weight (B) were analyzed weekly. Body weight gain (C) was measured from the 4^th^ week until the 17^th^ week in HF fed mice. Values are means±SEM, n = 8 for all groups. *Significantly different from the control and ASE groups (p≤.05). ^+^Significantly different from the corresponding HF group (p≤.05). ^#^Significantly different from the control and ASE groups (p≤.05).

The amount of food consumed (grams) was higher (*p*<0.05) in HF compared to control and ASE groups and the treatment with ASE prevented the increase of food intake in HF group (Fig 3A).

We compared male mice from the 4 groups with initial body weights at the age of 5 weeks and at the end of experimental protocol at the age of 17 weeks (Fig 3B). There was no body weight difference among the animals at the beginning of the study. However, from the third week to the end of the experimental protocol, the body weight gain of the HF group significantly increased (*p*<0.05, n = 8) compared to other groups (Fig 3C). Body weight of the HF+ASE group was reduced (*p*<0.05) compared to HF group, indicating a protective effect of ASE against overweight (Table 2).

**Table 2:** Effects of HF diet and ASE on body mass, food intake, energy intake, visceral adipose mass and liver parameters of C57BL/6 mice

Variables	Control	ASE	HF	HF+ASE
Final body mass (g)	24.1 ± 0.6	23.6 ± 0.6	42.8 ± 0.7*	31.4 ± 0.8* ^†^
Intake (g)	2.7 ± 0.1	2.8 ± 0.1	3.5 ± 0.1*	2.9 ± 0.1^†^
Energy intake (kcal)	10.3 ± 0.2	10.4 ± 0.2	17.4 ± 0.4*	14.6 ± 0.6* ^†^
*Fat mass (g)*				
Epididymal	0.66 ± 0.09	0.59 ± 0.09	1.99 ± 0.11*	1.35 ± 0.11* ^†^
Retroperitoneal	0.48 ± 0.13	0.36 ± 0.12	2.16 ± 0.15*	1.45 ± 0.13* ^†^
Liver/body weight (g)	0.046±0.01	0.041± 0.01	0.028 ± 0.01*	0.036 ± 0.01^†^

Data are means ± SEM, n = 8 for all groups.

*Significantly different from the Control and Control+ASE groups (p< 0.05).

^†^Significantly different from the corresponding HF group (p< 0.05).

### Inhibitory effect of ASE on adipokine levels, adipose and liver mass gain

Treatment with ASE reduced the epididymal and retroperitoneal adipose tissues weights from mice fed HF diet compared to mice fed HF diet (*p*<0.05, n = 8, Table 2). Liver weight in mice fed HF diet significantly increased compared to control mice, and ASE prevented liver weight gain in HF fed mice (*p*<0.05, Table 2).

HFD-induced obesity is also known to be strongly associated with the levels of adipokines such as leptin and adiponectin, both of which are secreted from adipose tissue. As shown in table 3, circulating plasma levels of leptin were increased in mice fed HF diet compared to control and ASE groups (*p*<0.05, n = 8), which reflects the increased fat deposits in HF mice. Treatment with ASE reduced plasma leptin levels in HF mice (*p*<0.05), indicating restoration of leptin sensitivity. In contrast, plasma levels of adiponectin were decreased in mice fed HF diet compared to control and ASE groups (*p*<0.05, n = 8). Treatment with ASE prevented the decrease in plasma adiponectin levels in HF mice (*p*<0.05).

**Table 3:** Effects of HF diet and ASE on glycaemia, plasma adipokines, serum and hepatic lipid profile in C57BL/6 mice

Variables	Control	ASE	HF	HF+ASE
Initial glucose (mg/dl)	55.4 ± 8.1	155.2 ± 7.2	164.1 ± 3.7	158.7 ± 0.2
Final glucose (mg/dl)	147.8 ± 5.9	142.6 ± 3.8	207.1 ± 4.5*	174.2 ± 3.5* ^†^
*Plasma*				
Leptin (ng/ml)	0.99 ± 0.17	0.96 ± 0.09	1.59 ± 0.01*	0.70 ± 0.21*
Adiponectin (ng/ml)	13.1 ± 0.7	13.3 ± 0.7	10.9 ± 0.3*	14.8 ± 0.2^†^
*Serum*				
TC (mg/dl)	98.6 ± 7.6	85.8 ± 8.9	180.6 ± 7.6*	132.7 ± 9.8* ^†^
TG (mg/dl)	28.3 ± 4,9	30.1 ± 3.4	97.7 ± 6.5 *	53.2 ± 3.1* ^†^
LDL	49.4 ± 5.6	49.2 ± 3.8	139.0 ± 1.6*	82.2 ± 2.2^†^
VLDL	5.6 ± 1.0	6.0 ± 0.7	19.5 ± 1.2*	10.6 ± 0.6* ^†^
*Liver*				
Cholesterol (mg/dl)	10.7 ± 1.8	12.2 ± 2.7	34.05 ± 4.2*	15.8 ± 1.5^†^
TG (mg/dl)	194.6 ± 46.9	115.8 ± 10.7	778.4 ± 26.8*	260.1 ± 52.7* ^†^

Data are means ± SEM, n = 8 for all groups.

*Significantly different from the Control and Control+ASE groups (p< 0.05).

^†^Significantly different from the corresponding HF group (p< 0.05).

### Effect of ASE on glycaemia, lipid profile and hepatic steatosis

Final blood glucose levels differed significantly between HF groups and controls. HF group developed hyperglycemia, whereas control and ASE groups remained normoglycemic at 17 weeks of age. Treatment of HF mice with ASE reduced (*p*<0.05) the hyperglycemia (n = 8, Table 3).

**Fig 4:**
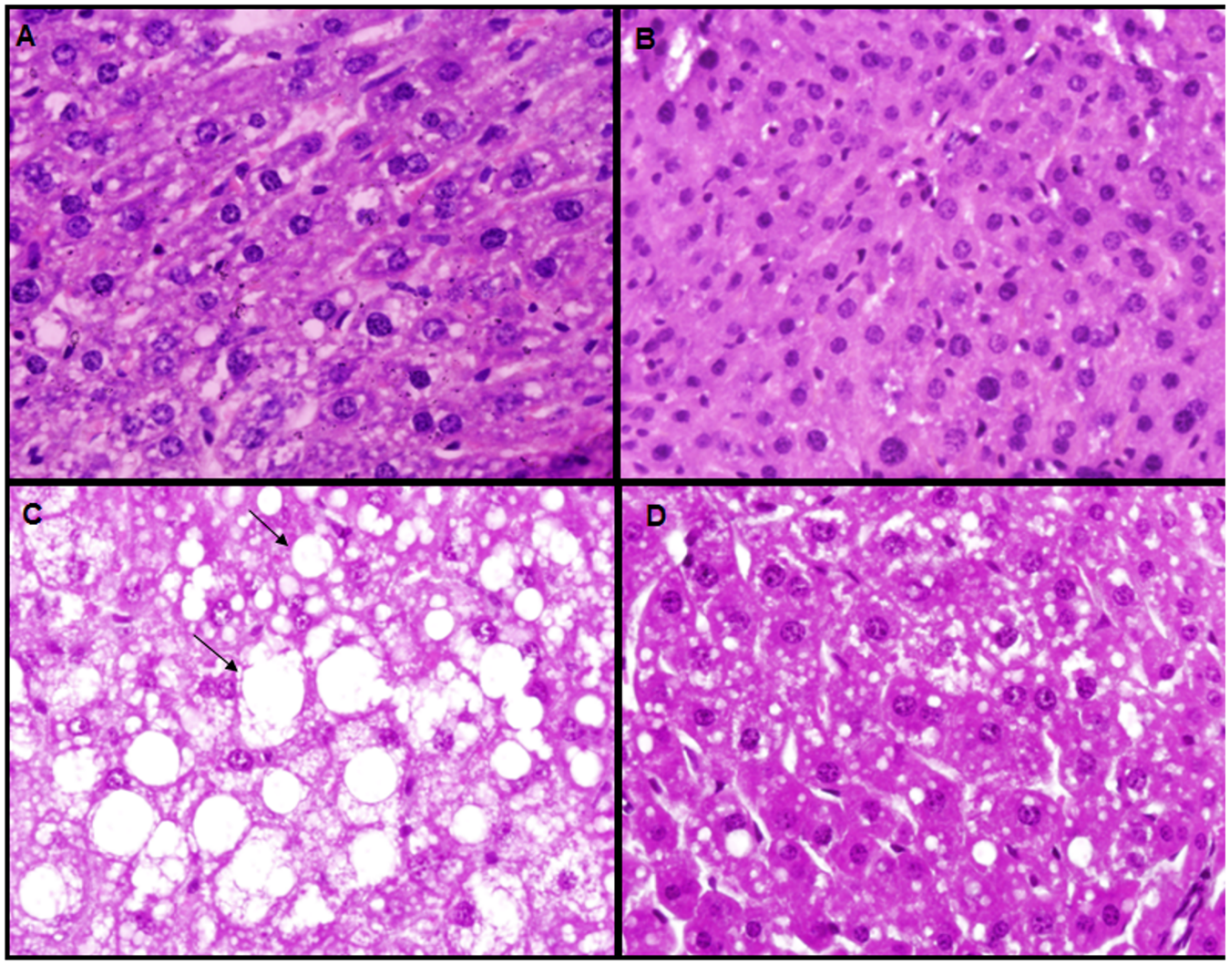
Photomicrographs of the liver structure. Sections of the liver were stained with hematoxylin and eosin, and each photomicrograph is shown at the same magnification. (A) The usual liver appearance in the control group; (B) ASE did not change the liver appearance; (C) macro- and micro-vesicular steatosis in the HF group; (D) steatosis was reduced in the HF+ASE group.

HF group showed macro- and microvesicular steatosis within hepatocytes. HF+ASE group showed less steatosis compared to untreated HF group (Fig 4). The serum levels of TC and TG were higher in the HF group compared to other groups at the end of study period (*p*<0.05, n = 8). Treatment with ASE reduced TC, TG, LDL and VLDL levels (Table 3).

The liver cholesterol and TG levels were higher (*p*<0.05) in HF group compared to control group (n = 8, Table 3). ASE significantly reduced (*p*<0.05) the liver cholesterol and TG levels in HF+ASE group compared to control group. There was no significant difference between control and ASE groups.

### Effect of ASE on the expression of proteins involved in fatty acid and cholesterol synthesis and excretion in liver

**Fig 5:**
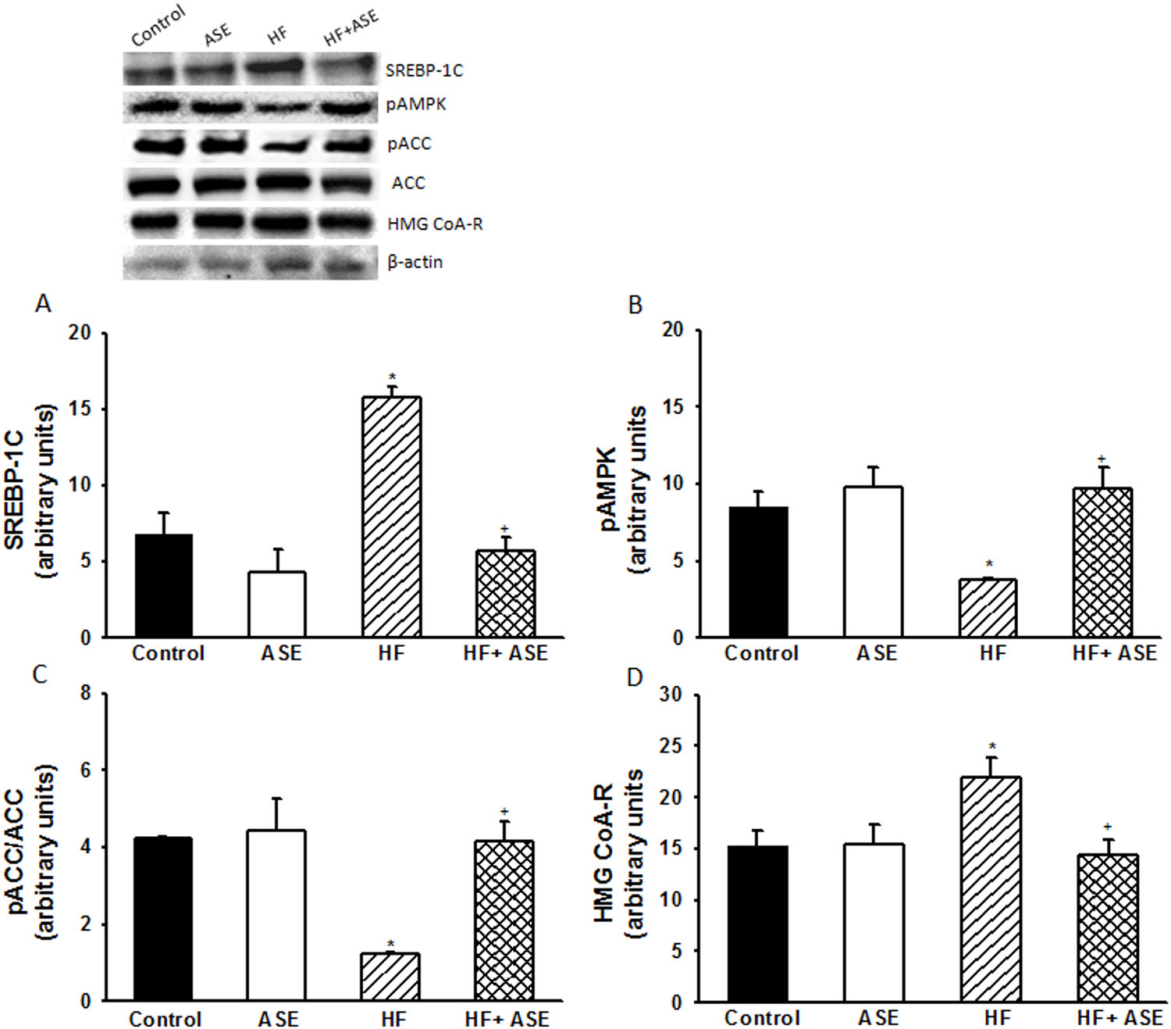
Expression of lipogenic proteins. Effects of ASE on SREBP-1 (A), pAMPK (B), pACC/ACC (C) and HMG CoA-R expressions in liver from HF fed mice. Values are means±SEM, n = 3 for all groups. *Significantly different from the control and ASE groups (p≤.05). ^+^Significantly different from the corresponding HF group (p≤.05).

To investigate the molecular mechanisms involved in the hypolipidemic effect of ASE, the expressions of SREBP1-c, p-ACC, ACC, HMG-CoA reductase and p-AMPK were assessed in the liver of the different groups. As shown in Fig 5, the HF group presented a reduction in the expression of pAMPK and in the ratio of pACC/ACC relative to the controls (*p*<0.05, n = 3). The treatment with ASE restored the AMPK phosphorylation and the ratio of pACC/ACC in HF group (*p*<0.05). The HF group showed an increase in the expression of HMG-CoA reductase relative to the control (*p*<0.05), which was impaired by ASE. In the untreated HF group, the expression of SREBP-1c was increased (*p*<0.05) compared to control mice and treatment with ASE prevented the increase (*p*<0.05) in SREBP-1c expression in HF fed mice (Fig 5).

**Fig 6:**
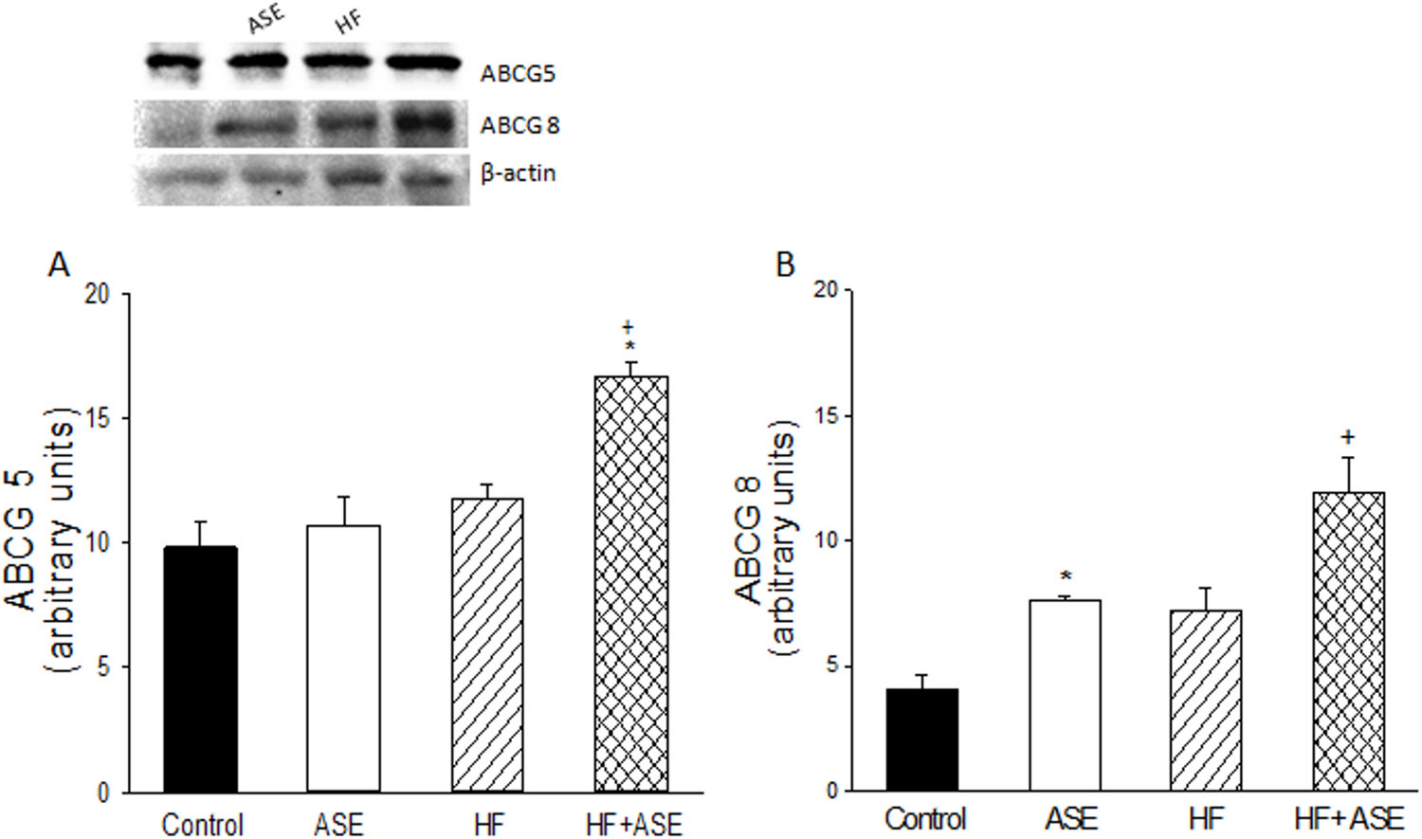
ABCG5 and ABCG8 transporters expression. Effect of ASE on ABCG5 (A) and ABCG8 (B) transporters expressions in liver from HF fed mice. Values are means±SEM, n = 3 for all groups. ^&^Significantly different from the control group (p≤.05). *Significantly different from the control and ASE groups (p≤.05). ^+^Significantly different from the corresponding HF group (p≤.05).

ASE increased the expressions of ABCG5 and ABCG8 transporters in HF fed mice (*p*<0.05) in comparison with other groups (Fig 6).

### Effect of ASE on hepatic oxidative damage and antioxidant activity

**Fig 7:**
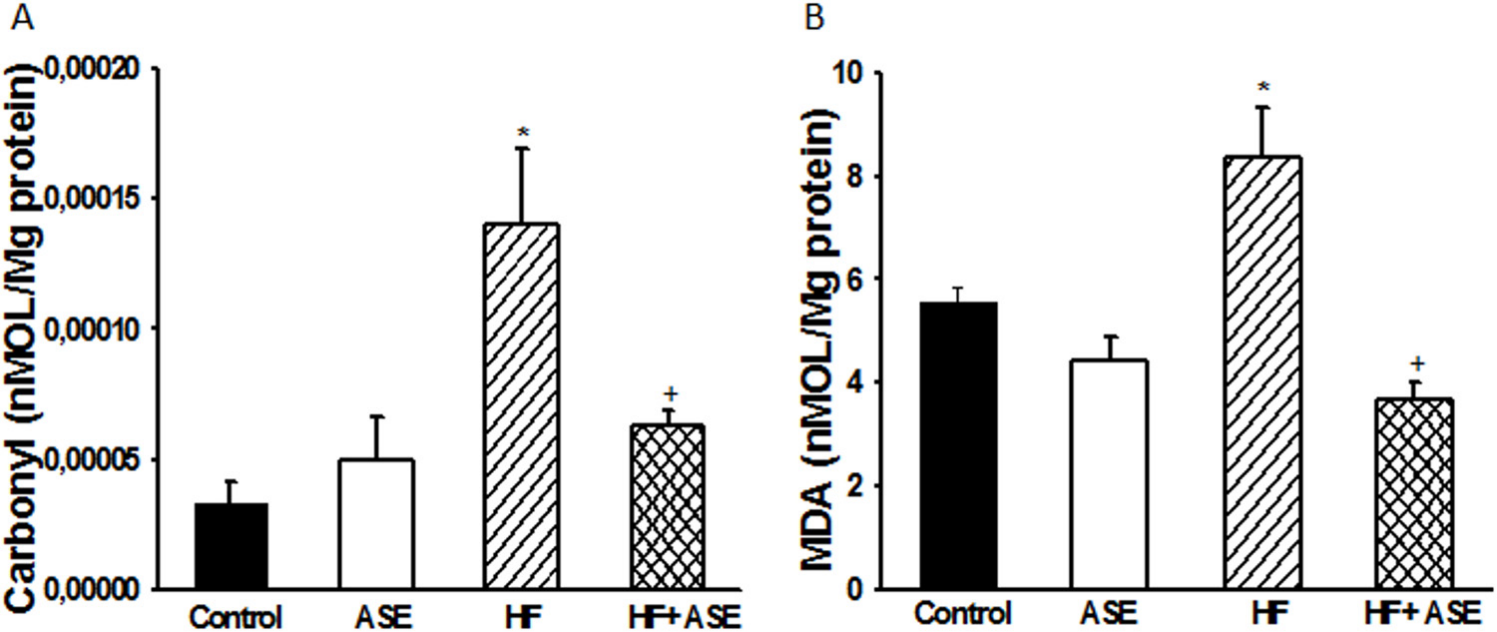
Protein carbonylation and malondialdehyde levels. Effects of ASE on protein carbonylation (A) and malondialdehyde levels (B) in liver from HF fed mice. Values are means±SEM, n = 8 for all groups. *Significantly different from the control and ASE groups (p≤.05). ^+^Significantly different from the corresponding HF group (p≤.05).

We observed a significant increase (*p*<0.05, n = 8) in the formation of by-products of lipid peroxidation (MDA) and carbonyl protein levels in HF group as compared to control animals (Fig 7). Treatment with ASE prevented the increase in MDA and carbonyl protein levels (*p*<0.05) in HF fed mice (Fig 7).

**Fig 8:**
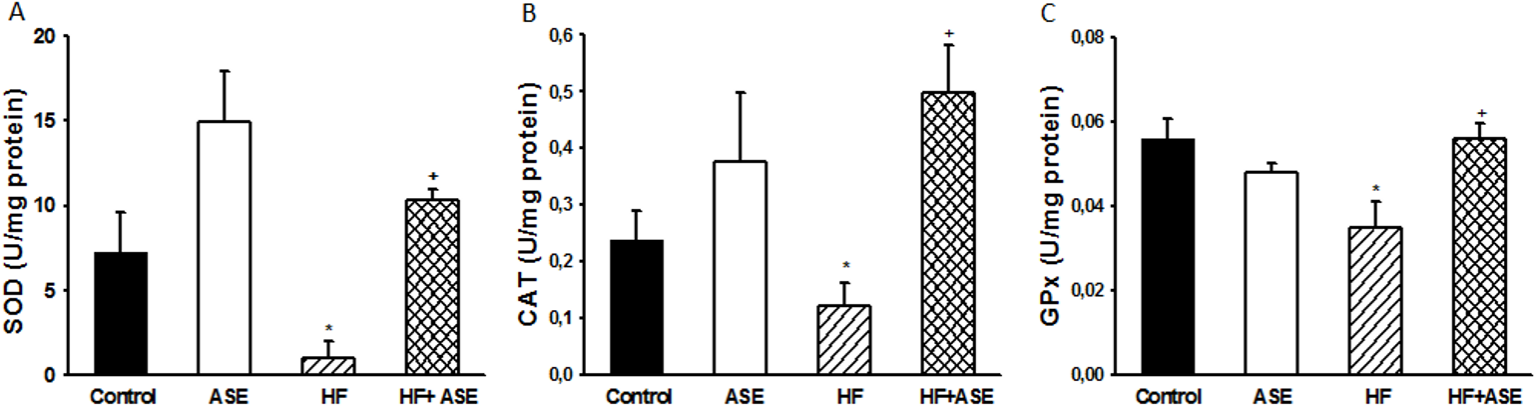
Antioxidant enzyme activities. Effects of ASE on superoxide dismutase (A), catalase (B) and glutathione peroxidase (C) activities in liver from HF fed mice. Values are means±SEM, n = 8 for all groups. *Significantly different from the control and ASE groups (p≤.05). ^+^Significantly different from the corresponding HF group (p≤.05).

The antioxidant enzyme activities (SOD, CAT and GPx) were reduced (*p*<0.05) in the liver homogenate of the HF group (Fig 8), as compared to the control groups, which was prevented by treatment with ASE (*p*<0.05).

## Discussion

MS and NAFLD are a major cause of morbidity in developed and developing societies [1, 31]. Insulin resistance and an unfavorable utilization of FA increase the hepatic content of TG and promote hepatic steatosis. Oxidative stress is one of the mechanisms preventing nontoxic degradation of excess lipids and promoting steatohepatitis. In the present study, we demonstrated that administration of ASE to mice fed a HF diet prevented the increase in body weight gain and significantly improved plasma and tissue metabolic profile, and hepatic steatosis to a similar metabolic condition comparable to mice fed a normal diet. The mechanisms underlying the beneficial effects of ASE involve the reduction of lipogenesis, increase in cholesterol excretion and improvement of oxidative stress in the liver.

Phenotypic changes, such as, increased adiposity, hyperglycemia, hyperlipidemia and hepatic steatosis are similar to previous studies in the same model [19, 32]. In this study, treatment with ASE substantially reduced the body weight gain and adiposity in obese mice from HF group. Adipose and liver masses correlated with body weight changes in mice treated with ASE, suggesting that the reduction in adipose and liver masses may contribute to ASE-mediated decrease in body weight. The possible mechanisms behind this antiobesity effect of ASE may be due to the presence of polyphenols in ASE [33] since it has been reported that polyphenols from other sources reduce serum TG, cholesterol and glucose [34, 35].

Adiponectin is one of the most important adipocytokines and is highly specific to adipose tissue [36]. We found a decreased plasma adiponectin concentration, which is in accordance with previous study in the same experimental model [32]. Evidence has shown that mice fed a high fat diet, develop hypoadiponectinemia associated with reduced activation of the critical energy sensor, AMPK and its target, ACC [37], as observed in the present study. Since AMPK activation is a cellular response to activate oxidative phosphorylation, reduced adiponectin may act via blunted cellular energy sensing mechanisms [37]. In the present study, treatment of mice fed a high fat diet with ASE prevented the hypoadiponectinemia associated with increased expression of pAMPK and pACC/ACC, indicating a role of increased adiponectin sensitivity to the ASE-mediated decrease in body weight. Previous studies have demonstrated that anthocyanins induce adiponectin gene expression in adipocytes from C57BL/6 fed a HF diet [38]. In addition, treatment with ASE reduced plasma leptin levels in HF fed mice, indicating restoration of leptin sensitivity leading to the reduction of food intake and therefore, low fat accumulation.

The prevalence of hyperlipidemia in humans with NAFLD ranges from 20 to 92% [39]. It is widely accepted that nutritional excess, mainly through the consumption of a diet high in saturated fat, can cause liver damage by the accumulation of cholesterol and TG. In the current study, a significant reduction in both serum and liver TG and cholesterol was observed in mice fed a HF diet and treated with ASE. Similarly, some human and animal studies demonstrated that Açaí has a positive effect in lowering serum cholesterol and TG [19, 40], suggesting a lipid-lowering action to Açaí.

Removal of the excess of cholesterol from the body is essential for the maintenance of homeostasis. The ABCG transporter ABCG8 is responsible for the efflux of cholesterol in the bile [41]. Açaí pulp was recently shown to produce a hypocholesterolemic effect in rats fed a high cholesterol diet, an effect mediated by an over-expression of the ABCG5 and ABCG 8 transporters, resulting in a profound cholesterol trafficking which is characterized by an increase in the biliary cholesterol secretion and a decrease in cholesterol absorption [41]. In the present study, we found that ASE increased the expressions of ABCG5 and ABCG8 transporters in the liver of obese mice. Up-regulation of these transporters is the likely mechanism underlying the decreased concentration of serum and liver cholesterol.

HMG-CoA reductase is the key enzyme in the regulation of cholesterol synthetic pathway. The activity of this enzyme is regulated by AMPK, which in its phosphorylated and active state (pAMPK), promotes inactivation of HMG-CoA reductase [5]. Once AMPK is activated, lipogenesis in liver is inhibited, which consequently inhibits fat accumulation [5; 42]. HF diet inhibits AMPK phosphorylation in C57BL/6 mice [43, 44]. A recent study in mice fed a diet with 60% fat for 5 weeks, showed a significant increase in HMG-CoA reductase expression and activity, which correlated with accumulation of cholesterol in the liver [45]. In this study, the expression of HMG-CoA reductase was increased in the liver from HF fed mice and that of pAMPK was reduced, which is in agreement with previews findings in the same experimental model [42]. These data suggest that the negative modulation exerted by pAMPK on cholesterol synthesis is compromised in obese animals, resulting in increased cholesterol levels observed in this study. We demonstrated for the first time that ASE impaired the reduction of pAMPK expression and the increase of HMG-CoA reductase in the liver of mice fed a HF diet. These findings suggest an important modulation by ASE of AMPK pathway involved in the regulation of body weight, lipid metabolism and glucose homeostasis [4].

The hepatic steatosis observed in this study is characterized by increased lipid (mainly TG) levels in the liver. SREBP-1c plays a unique role in the expression of the genes involved in hepatic TG synthesis and, may play a major role in the pathogenesis of NAFLD [46]. The lipogenic genes regulated by SREBP-1C, include ACC that converts acetyl-CoA substrate in malonyl-CoA. Subsequently, malonyl-CoA is converted into palmitate and FA that can be esterified to TG [46]. A positive energy imbalance, characteristic of this model, chronically activates SREBP1-c, causing lipotoxicity in various organs and tissues. Insulin seems to play a central role in the activation of SREBP-1c transcription, even with the presence of insulin resistance [47]. Furthermore, liver pAMPK is known to regulate the synthesis of FA and TG, by inactivating ACC [48]. In the present study, we observed an increased expression of the pro-lipogenic transcription factor SREBP-1c protein and reduced the ratio of pACC/ACC expression in HF fed mice, indicating the induction of lipogenesis, which is in agreement with previews findings in the same experimental model [32,42]. ASE significantly reduced the SREBP-1c expression and increased the ratio of pACC/ACC to the normal levels, which was associated with reduction in TG levels and effective reduction of hepatic steatosis. These findings suggest a role to ASE in the control of endogenous FA synthesis, accumulation of lipids and lipotoxicity.

Another important aspect of the regulation of lipid metabolism is the liver mitochondrial oxidative function. Several studies in animal models indicate a significant relationship between accumulation of TG, mitochondrial dysfunction and oxidative stress [49]. It is conceivable that hyperglycemia and high levels of saturated FA can promote greater activation of NADPH oxidase and consequently higher reactive oxygen species (ROS) generation [50]. Mitochondria continuously exposed to high levels of ROS can suffer deleterious consequences, such as damage to the respiratory chain in the mitochondrial genome and in lipids and membrane proteins [51]. These structural and functional alterations in mitochondria may contribute to the reduction of the activities of this organelle as beta oxidation leading to a greater accumulation of free FA [52]. Associated with this process there is a lower formation of antioxidant enzymes [50] leading to an imbalance between ROS formation and antioxidant protection featuring oxidative stress.

In the current study, we found a significant increase in MDA and protein carbonyl levels, in the liver of mice fed by HF diet which may be related to mitochondrial dysfunction and may contribute to the accumulation of fat in the liver of these animals [50]. Furthermore, a significant decrease in hepatic antioxidant enzyme activities was detected in the mice fed a HF diet. Treatment with ASE markedly decreased the MDA and protein carbonylation levels and restored antioxidant activity of SOD, CAT and GPx to levels similar to controls fed by standard diet. A study by de Oliveira et al showed that high levels of plasma MDA in mice fed by high fat diet were reduced in response to ASE [19]. Guerra et al demonstrated that 2% Açaí extract added to the diet of control and diabetic rats increased the total hepatic content of glutathione and increased mRNA expression of different liver anti-oxidant enzymes including GPx [53]. Catechin and polymeric proanthocyanidins may be the major contributors to the antioxidant activity of ASE.

## Conclusions

The present study demonstrated that in HF-fed mice ASE decreased food intake and body mass gain, and ameliorates both adiposity and hepatic steatosis. The underlying mechanisms may involve reduction in hepatic lipogenesis, which can primarily be attributed to reduced expression of SREBP-1c and increased expression of pAMPK, which negatively modulates ACC and HMG-CoA reductase. The beneficial effect of ASE can be due to an increase in cholesterol excretion by ABCG8 transporter, as well as the antioxidant effect in liver.

## Abbreviations

ASE: açaí seed extract
MS: metabolic syndrome
NAFLD: nonalcoholic fatty liver disease
HF: high fat
TG: triacyglycerol
AMPK: adenosine-monophosphate-activated protein kinase
pAMPK: phosphorilated adenosine-monophosphate-activated protein kinase
SREBP-1c: sterol-regulatory-element binding protein-1c
ACC: acetyl-CoA carboxylase
pACC: phosphorilated acetyl-CoA carboxylase
HMG-CoA reductase: 3-hydroxy-3-methylglutaryl CoA reductase
ABCG8: ATP-biding cassette, subfamily G transporter 8
MDA: malondialdehyde
SOD: superoxide dismutase
CAT: catalase
GPx: glutathione peroxidase

## References

[1] Asrih M, Jornayvaz FR. Metabolic syndrome and nonalcoholic fatty liver disease: Is insulin resistance the link? Mol Cell Endocrinol. 2015;10(5): e0126364. 10.1016/j.mce.2015.02.018 25724480

[2] Cao K, Xu J, Zou X, Li Y, Chen C, Zheng A, et al Hydroxytyrosol prevents diet inducedmmetabolic syndrome and attenuates mitochondrial abnormalities in obese mice. Free Radic Biol Med. 2014;67: 396–407. 10.1016/j.freeradbiomed.2013.11.029 24316371

[3] Kabir M, Catalano KJ, Ananthnarayan S, Kim SP, Van Citters GW, Dea MK, et al Molecular evidence supporting the portal theory: a causative link between visceral adiposity and hepatic insulin resistance. Am J Physiol Endocrinol Metab. 2005;288(2): E454–E461. 15522994 10.1152/ajpendo.00203.2004

[4] Kahn BB, Alquier T, Carling D, Hardie DG. AMP-activated protein kinase: ancient energy gauge provides clues to modern understanding of metabolism. Cell Metab. 2005;1(1): 15–25. 16054041 10.1016/j.cmet.2004.12.003

[5] Hardie DG, Corton J, Ching YP, Davies SP, Hawley S. Regulation of lipid metabolism by the AMP-activated protein kinase. Biochem Soc Trans 1997; 25(4): 1229–1231. 9449981 10.1042/bst0251229

[6] Kohjima M, Higuchi N, Kato M, Kotoh K, Yoshimoto T, Fujino T, et al SREBP-1c, regulated by the insulin and AMPK signaling pathways, plays a role in nonalcoholic fatty liver disease. Int J Mol Med. 2008;21(4): 507–511. 18360697

[7] Yu L, Li-Hawkins J, Hammer RE, Berge KE, Horton JD, Cohen JC, et al Overexpression of ABCG5 and ABCG8 promotes biliary cholesterol secretion and reduces fractional absorption of dietary cholesterol. J Clin Invest. 2002; 110:671–680. 12208868 10.1172/JCI16001 PMC151111

[8] Ceriello A, Motz E. Is oxidative stress the pathogenic mechanism underlying insulin resistance, diabetes, and cardiovascular disease? The common soil hypothesis revisited. Arterioscler Thromb Vasc Biol. 2004;24(5): 816–823. 14976002 10.1161/01.ATV.0000122852.22604.78

[9] Leung TM, Nieto N: CYP2E1 and oxidant stress in alcoholic and non-alcoholic fatty liver disease. J Hepatol. 2013;58: 395–398. 10.1016/j.jhep.2012.08.018 22940046

[10] Nagata K, Suzuki H, Sakaguchi S: Common pathogenic mechanism in development progression of liver injury caused by non-alcoholic or alcoholic steatohepatitis. J Toxicol Sci. 2007;32: 453–468. 18198478 10.2131/jts.32.453

[11] Sanyal AJ, Chalasani N, Kowdley KV, McCullough A, Diehl AM, Bass NM, et al: Pioglitazone, vitamin E, or placebo for nonalcoholic steatohepatitis. N Engl J Med 2010;362: 1675–1685. 10.1056/NEJMoa0907929 20427778 PMC2928471

[12] Mazza A, Fruci B, Garinis GA, Giuliano S, Malaguarnera R, Belfiore A: The role of metformin in the management of NAFLD. Exp Diabetes Res. 2012;2012: 716404 10.1155/2012/716404 22194737 PMC3238361

[13] Ratziu V, Charlotte F, Bernhardt C, Giral P, Halbron M, Lenaour G, et al Long-term efficacy of rosiglitazone in nonalcoholic steatohepatitis: results of the fatty liver improvement by rosiglitazone therapy (FLIRT 2) extension trial. Hepatology 2010;51: 445–453. 10.1002/hep.23270 19877169

[14] McCarthy EM, Rinella ME: The role of diet and nutrient composition in nonalcoholic Fatty liver disease. J Acad Nutr Diet 2012;112: 401–409. 10.1016/j.jada.2011.10.007 22717200

[15] Schauss AG, Wu X, Prior RL, Ou B, Huang D, Owens J, et al Antioxidant capacity and other bioactivities of the freeze-dried Amazonian palm berry, Euterpe oleraceae mart. (acai). J Agric Food Chem. 2006; 54: 8604–8610. 17061840 10.1021/jf0609779

[16] Moura RS, Ferreira TS, Lopes AA, Pires KM, Nesi RT, Resende AC, et al Effects of Euterpe oleracea Mart. (Açaí) extract in acute lung inflammation induced by cigarette smoke in the mouse. Phytomedicine 2012;19(3–4): 262–269. 10.1016/j.phymed.2011.11.004 22138278

[17] Rocha AP, Carvalho LC, Sousa MA, Madeira SV, Sousa PJ, Tano T, et al Endothelium-dependent vasodilator effect of Euterpe oleracea Mart. (Acai) extracts in mesenteric vascular bed of the rat. Vascul Pharmacol. 2007;46(2): 97–104. 17049314 10.1016/j.vph.2006.08.411

[18] da Costa CA, de Oliveira PR, de Bem GF, de Cavalho LC, Ognibene DT, da Silva AF, et al Euterpe oleracea Mart.-derived polyphenols prevent endothelial dysfunction and vascular structural changes in renovascular hypertensive rats: role of oxidative stress. Naunyn Schmiedebergs Arch Pharmacol. 2012;385(12): 1199–1209. 10.1007/s00210-012-0798-z 23052352

[19] Oliveira PR, da Costa CA, de Bem GF, de Cavalho LCRM, de Souza MA, de Lemos Neto et al. Effects of an extract obtained from fruits of Euterpe oleracea Mart. in the components of metabolic syndrome induced in C57BL/6J mice fed a high-fat diet. J Cardiovasc Pharmacol. 2010;56(6): 619–626. 10.1097/FJC.0b013e3181f78da4 20838232

[20] Peng Z, Hayasaka Y, Iland PG, Sefton M, Høj P, Waters EJ. Quantitative analysis of polymeric procyanidins (Tannins) from grape (Vitis vinifera) seeds by reverse phase high-performance liquid chromatography. J Agric Food Chem. 2001;49(1): 26–31. 11170555 10.1021/jf000670o

[21] Kennedy JA, Jones GP. Analysis of proanthocyanidin cleavage products following acid-catalysis in the presence of excess phloroglucinol. J Agric Food Chem. 2001; 49:1740–1746. 11308320 10.1021/jf001030o

[22] Kelm MA, Johnson JC, Robbins RJ, Hammerstone JF, Schmitz HH. High-performance liquid chromatography separation and purification of cacao (*Theobroma cacao* L.) procyanidins according to degree of polymerization using a diol stationary phase. J Agric Food Chem. 2006;54: 1571–1576. 16506802 10.1021/jf0525941

[23] Mateos-Martín ML, Fuguet E, Quero C, Pérez-Jiménez J, Torres JL. New identification of proanthocyanidins in cinnamon (*Cinnamomum zeylanicum* L.) using MALDI-TOF/TOF mass spectrometry. Anal Bioanal Chem. 2012; 402: 1327–1336. 10.1007/s00216-011-5557-3 22101466

[24] Reeves PG, Nielsen FH, Fahey GC. AIN-93 purified diets for laboratory rodents: final report of the American Institute of Nutrition ad hoc writing committee on the reformulation of the AIN-76A rodent diet. J Nutr. 1993;123: 1939–1951. 8229312 10.1093/jn/123.11.1939

[25] Levine RL, Garland D, Oliver CN, Amici A, Climent I, Lenz AG, et al Determination of carbonyl content in oxidatively modified proteins. Meth Enzymol. 1990;186: 464–478. 1978225 10.1016/0076-6879(90)86141-h

[26] Bannister JV, Calabrese L. Assays for superoxide dismutase. Methods Biochem Anal. 1987;32: 279–312. 3033431 10.1002/9780470110539.ch5

[27] Aebi H. Catalase in vitro. Meth Enzymol. 1984;105: 121–126. 6727660 10.1016/s0076-6879(84)05016-3

[28] Flohe L, Gunzler WA. Assays of glutathione peroxidase. Methods Enzymol. 1984;105: 114–121. 6727659 10.1016/s0076-6879(84)05015-1

[29] Monagas M, Quintanilla-López JE, Gómez-Cordovés C, Bartolomé B, Lebrón-Aguilar R. MALDI-TOF MS analysis of plant proanthocyanidins. J Pharmaceutical and Biomedical Anal. 2010;51: 358–372. 10.1016/j.jpba.2009.03.035 19410413

[30] Stringano E, Cramer R, Hayes W, Smith C, Gibson T, Mueller-Harvey I. Deciphering the complexity of sainfoin (Onobrychis viciifolia) proanthocyanidins by MALDI-TOF mass spectrometry with a judicious choice of isotope patterns and matrixes. Anal Chem. 2011;83(11): 4147–41. 10.1021/ac2003856 21488615

[31] Jung UJ, Choi MS.Obesity andits metabolic complications:the role of adipokines and the relationship between obesity,inflammation, insulin resistance, dyslipidemia and nonalcoholic fatty liver disease. Int J Mol Sci. 2014;15(4): 6184–223. 10.3390/ijms15046184 24733068 PMC4013623

[32] Fraulob JC, Souza-Mello V, Aguila MB, Mandarim-de-Lacerda CA. Beneficial effects of rosuvastatin on insulin resistance, adiposity, inflammatory markers and non-alcoholic fatty liver disease in mice fed on a high-fat diet. Clin Sci. 2012;123(4): 259–270. 10.1042/CS20110373 22420611

[33] Rodrigues RB, Lichtenthaler R, Zimmermann BF, Papagiannopoulos M, Fabricius H, Marx F et al Total oxidant scavenging capacity of Euterpe oleracea Mart. (acai) seeds and identification of their polyphenolic compounds. J Agric Food Chem. 2006; 54:4162–4167. 16756342 10.1021/jf058169p

[34] Snoussi C, Ducroc R, Hamdaoui MH, Dhaouadi K, Abaidi H, Cluzeaud F, et al Green tea decoction improves glucose tolerance and reduces weight gain of rats fed normal and high-fat diet. J Nutr Biochem. 2014;25(5):557–564. 10.1016/j.jnutbio.2014.01.006 24656388

[35] Chiva-Blanch G, Urpi-Sarda M, Ros E, Valderas-Martinez P, Casas R, Arranz S, Guillén M, et al Effects of red wine polyphenols and alcohol on glucose metabolism and the lipid profile: a randomized clinical trial. Clin Nutr. 2013;32(2): 200–206. 10.1016/j.clnu.2012.08.022 22999066

[36] Combs TP, Marliss EB. Adiponectin signaling in the liver Rev Endocr Metab Disord. 2013 10.1007/s11154-013-9280-6 PMC4152934 24297186

[37] Iwabu M, Yamauchi T, Okada-Iwabu M, Sato K, Nakagawa T, Funata M, et al Adiponectin and AdipoR1 regulate PGC-1alpha and mitochondria by Ca(2+) and AMPK/SIRT1. Nature. 2010;464(7293): 1313–1319. 10.1038/nature08991 20357764

[38] Tsuda T, Ueno Y, Aoki H, Koda T, Horio F, Takahashi N, et al Anthocyanin enhances adipocytokine secretion and adipocyte-specific gene expression in isolated rat adipocytes. Biochem Biophys Res Commun. 2004;316(1): 149–157. 15003523 10.1016/j.bbrc.2004.02.031

[39] Angulo P: Nonalcoholic fatty liver disease. N Engl J Med. 2002;346: 1221–1231. 11961152 10.1056/NEJMra011775

[40] Udani JK, Singh BB, Singh VJ, Barrett ML. Effects of Acai (Euterpe oleracea Mart.) berry preparation on metabolic parameters in a healthy overweight population: a pilot study. Nutr J. 2011; 10: 45 10.1186/1475-2891-10-45 21569436 PMC3118329

[41] de Souza MO, Souza ESL, de Brito Magalhaes CL, de Figueiredo BB, Costa DC, Silva ME, et al The hypocholesterolemic activity of acai (Euterpe oleracea Mart.) is mediated by the enhanced expression of the ATP-binding cassette, subfamily G transporters 5 and 8 and low-density lipoprotein receptor genes in the rat. Nutr Res. 2012;32: 976–984. 10.1016/j.nutres.2012.10.001 23244543

[42] Choi KM, Lee YS, Shin DM, Lee S, Yoo KS, Lee MK, Lee et al Green tomato extract attenuates high-fat-diet-induced obesity through activation of the AMPK pathway in C57BL/6 mice. J Nutr Biochem. 2013;24(1): 335–342. 10.1016/j.jnutbio.2012.06.018 22974972

[43] Wu CH, Yang MY, Chan KC, Chung PJ, Ou TT, Wang CJ. Improvement in high-fat diet-induced obesity and body fat accumulation by a Nelumbo nucifera leaf flavonoid-rich extract in mice. J Agric Food Chem. 2010;58(11): 7075–7081. 10.1021/jf101415v 20481471

[44] Seo JB, Choe SS, Jeong HW, Park SW, Shin HJ, Choi SM, et al Anti-obesity effects of Lysimachia foenum-graecum characterized by decreased adipogenesis and regulated lipid metabolism. Exp Mol Med. 2011;43(4): 205–215. 21389766 10.3858/emm.2011.43.4.025 PMC3085739

[45] Wu N, Sarna LK, Hwang SY, Zhu Q, Wang P, Siow YL, et al Activation of 3-hydroxy-3-methylglutaryl coenzyme A (HMG-CoA) reductase during high fat diet feeding. Biochim Biophys Acta 2013;1832(10): 1560–1568. 10.1016/j.bbadis.2013.04.024 23651731

[46] Dulloo AG, Gubler M, Montani JP, Seydoux J, Solinas G. Substrate cycling between de novo lipogenesis and lipid oxidation: a thermogenic mechanism against skeletal muscle lipotoxicity and glucolipotoxicity. Int J Obes Relat Metab Disord. 2004;28(4): S29–S37. 10.1038/sj.ijo.0802861 15592483

[47] Postic C, Girard J. Contribution of de novo fatty acid synthesis to hepatic steatosis and insulin resistance: lessons from genetically engineered mice. J Clin Invest. 2008;118(3): 829–838. 10.1172/JCI34275 18317565 PMC2254980

[48] Hou X, Xu S, Maitland-Toolan KA, Sato K, Jiang B, Ido Y, et al SIRT1 regulates hepatocyte lipid metabolism through activating AMP-activated protein kinase. J Biol Chem. 2008;283(29): 2015–2026. 10.1074/jbc.M802187200 PMC2459285 18482975

[49] Begriche K, Igoudjil A, Pessayre D, Fromenty B. Mitochondrial dysfunction in NASH: causes, consequences and possible means to prevent it. Mitochondrion. 2006;6(1): 1–28. 16406828 10.1016/j.mito.2005.10.004

[50] Carmiel-Haggai M, Cederbaum AI, Nieto N. A high-fat diet leads to the progression of non-alcoholic fatty liver disease in obese rats. FASEB J. 2005; 19(1): 136–138. 15522905 10.1096/fj.04-2291fje

[51] Ricci C, Pastukh V, Leonard J, et al Mitochondrial DNA damage triggers mitochondrial-superoxide generation and apoptosis. Am J Physiol 2008;294(2): C413–C422. 10.1152/ajpcell.00362.2007 18077603

[52] Rector RS, Thyfault JP, Uptergrove GM, Morris EM, Naples SP, Borengasser SJ, et al Mitochondrial dysfunction precedes insulin resistance and hepatic steatosis and contributes to the natural history of non-alcoholic fatty liver disease in an obese rodent model. J Hepatol 2010;52(5): 727–736. 10.1016/j.jhep.2009.11.030 20347174 PMC3070177

[53] Guerra JF, Magalhaes CL, Costa DC, Silva ME, Pedrosa ML. Dietary acai modulates ROS production by neutrophils and gene expression of liver antioxidant enzymes in rats. J Clin Biochem Nutr. 2011;49: 188–194. 10.3164/jcbn.11-02 22128218 PMC3208015

